# Covalent Organic Framework Membranes and Water Treatment

**DOI:** 10.1021/jacs.3c10832

**Published:** 2024-02-01

**Authors:** Muhammad Bilal Asif, Seokjin Kim, Thien S. Nguyen, Javeed Mahmood, Cafer T. Yavuz

**Affiliations:** † Oxide & Organic Nanomaterials for Energy & Environment (ONE) Laboratory, Chemistry Program, Physical Science & Engineering (PSE), King Abdullah University of Science and Technology (KAUST), Thuwal 23955, Saudi Arabia; ‡ Advanced Membranes & Porous Materials (AMPM) Center, Physical Science & Engineering (PSE), King Abdullah University of Science and Technology (KAUST), Thuwal 23955, Saudi Arabia; § KAUST Catalysis Center (KCC), Physical Science & Engineering (PSE), King Abdullah University of Science and Technology (KAUST), Thuwal 23955, Saudi Arabia

## Abstract

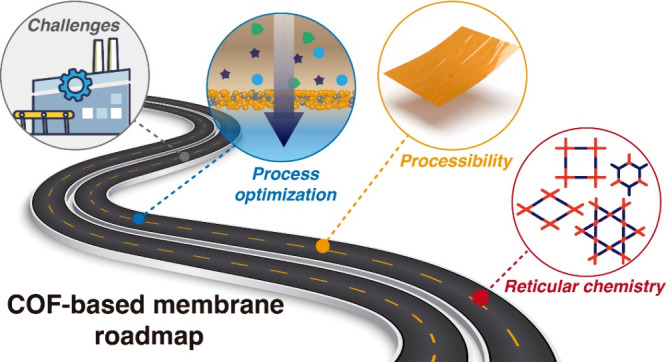

Covalent organic frameworks (COFs) are an emerging class of highly porous crystalline organic polymers comprised entirely of organic linkers connected by strong covalent bonds. Due to their excellent physicochemical properties (e.g., ordered structure, porosity, and stability), COFs are considered ideal materials for developing state-of-the-art separation membranes. In fact, significant advances have been made in the last six years regarding the fabrication and functionalization of COF membranes. In particular, COFs have been utilized to obtain thin-film, composite, and mixed matrix membranes that could achieve effective rejection (mostly above 80%) of organic dyes and model organic foulants (e.g., humic acid). COF-based membranes, especially those prepared by embedding into polyamide thin-films, obtained adequate rejection of salts in desalination applications. However, the claims of ordered structure and separation mechanisms remain unclear and debatable. In this perspective, we analyze critically the design and exploitation of COFs for membrane fabrication and their performance in water treatment applications. In addition, technological challenges associated with COF properties, fabrication methods, and treatment efficacy are highlighted to redirect future research efforts in realizing highly selective separation membranes for scale-up and industrial applications.

## 1 Introduction

Membrane separation is an established technology that is widely applied in pharmaceutical industries for sterile filtration, in the food industry for processing, and in the water industry for decontamination and desalination. Owing to the uneven distribution of water around the globe and the adversities brought up by climate change, water-stressed regions and coastal areas are required to meet their freshwater demand by seawater desalination and water reclamation using membrane technology.^1,2^ Compared to conventional separation or desalination processes (e.g., distillation), the advantages offered by membrane technology include compactness, robustness, ease of scalability, and energy efficiency.^1,2^ According to estimates, membrane technology has become an industry of around 24.65 billion US dollars in 2022.^3^ Since the installation of the first reverse osmosis (RO) membrane-based desalination plant in 1965, the quest to develop and improve the energy efficiency, cost-effectiveness, and stability of desalination membranes is still going on to this day.^1,2,4^ The total cumulative desalination capacity of the installed plants in 2020 was estimated to be 114.8 million m^3^ d^–1^ and has shown a steady upward trajectory with an annual increase of 7% from 2010 to 2019. Depending on the plant size and geographical location, membrane-based desalination costs from 0.14 $ m^–3^ to 2.46 $ m^–3^.^1^ Polyamide thin film nanocomposites (TFN) are considered as the “gold standard” for RO and nanofiltration (NF) processes in desalination due to their low cost, outstanding flexibility, and adequate mechanical strength. The performance of these membranes is assessed based on their permeability and selectivity, which generally show an inverse relationship; i.e., selectivity increases with a reduction in permeability and *vice versa*.^5^ Given the advances in material science for the discovery of new building blocks or polymers,^4,6−8^ new separation membranes are designed with the following design criteria: (i) uniform and ordered pore size; (ii) narrow pore size distribution; (iii) thin active layer; and (iv) affinity of permeant with membrane for high permeability.

Covalent organic frameworks (COFs) were first developed in 2005.^9^ COFs are a class of organic porous crystalline materials and are characterized by high surface area, high porosity, tunable pore size, and readily amenable surface properties.^7,8^ Due to these exceptional features, COFs have been considered ideal materials for the development of membranes with high permselectivity. The expectation is that the ordered structure of COFs would facilitate the precise sieving of molecules (via size exclusion) at high permeate flux. At the same time, their covalent linkage and crystalline nature would ensure their stability under harsh operating conditions. However, the poor dispersibility of COFs in solvents made their processability challenging, and consequently it took 12 years after their discovery to realize their first application in water treatment for organic dye rejection.^10^ This gave a much needed boost and opened a floodgate of studies on COF membranes. These studies are mainly focused on the membrane fabrication methods, including interface-assisted polymerization,^10,11^*in situ* growth,^12^ layer-by-layer stacking,^13^ blending,^14,15^ and incorporation into polyamide TFN membranes.^16−19^ In the meantime, intrinsic properties of COFs and membranes were played with and engineered by tuning the pore size, and adjusting the hydrophilicity with the intention to enhance desalination performance.^20−22^ In terms of water treatment, COF membranes have been predominantly used as NF membranes for organic dye rejection,^23−30^ whereas desalination performance with a salt rejection efficiency of 10–97% has been reported in the literature.^19,31−35^ Moreover, the potential of COF membranes for ultrafiltration (UF),^24,36,37^ forward osmosis (FO),^38,39^ and membrane distillation (MD)^40^ have also been explored.

The COF membranes have come a long way in just six years, and significant advances have been made, particularly in their synthesis and fabrication. However, research directions for COF membranes are unclear and seem to have less focus on the real-world problems of polymeric membranes. In this perspective, we aim to critically evaluate the current progress of COF membranes in water treatment and evaluate the accompanying technological challenges. The progress in the development of COFs has been reviewed previously.^7,8,41,42^ However, synthesis methods and COF membranes were mainly evaluated for broader applications such as gas separation,^8,41^ catalysis,^8^ adsorption,^7,8^ organic solvent nanofiltration,^41,42^ and water treatment.^41,42^ This perspective identifies the challenges that need urgent attention. First, we discuss the intrinsic properties of COFs and outline tuning/adjustment strategies along with implications in water treatment. Second, the synthesis of COF membranes using different methods is elucidated, followed by the challenges encountered during material processing that could affect their ability to fabricate high quality crystalline membranes. Third, a comprehensive evaluation of COF membrane performance for water treatment and desalination is carried out to provide insights into their permselectivity and scale-up potential. The applicability of reported performance and claimed separation mechanisms are also discussed. Finally, in addition to the research questions raised in each section, we summarized the overall challenges faced by COF membranes and the opportunities they will bring.

## 2 Engineering the Intrinsic Properties of COFs for Membranes

In general, intrinsic properties of the materials used for membrane fabrication govern their overall performance, including permselectivity and stability. For instance, as the size sieving is the primary separation mechanism in the majority of membrane-based separations, pore size is a key parameter in water treatment. In this section, we discuss the engineering of the intrinsic properties of COFs and their adjustments for the fabrication of membranes.

### 2.1 Pore Size Engineering

**Figure 1:**
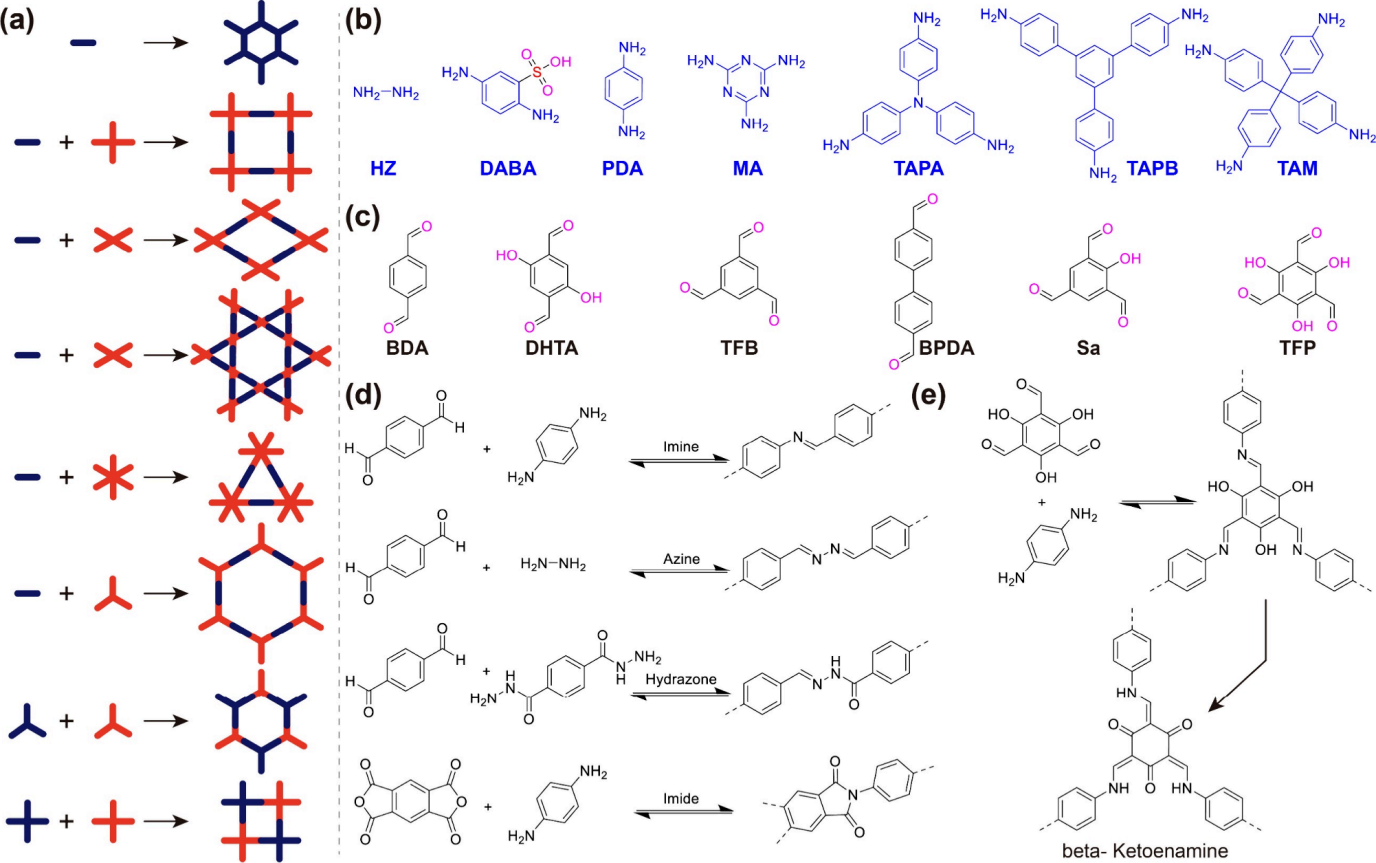
(a) Typical geometries of linkers and COF topologies. (b) Examples of commonly used amine monomers. (c) Examples of commonly used aldehyde monomers. (d, e) Examples of typical linkage bonds in COF membranes. HZ: Hydrazine; DABA: 2,5-diaminobenzenesulfonic acid; PDA: *p*-Phenylenediamine; MA: 1,3,5-triazine-2,4,6-triamine; TAPA: N1,N1-bis(4-aminophenyl)benzene-1,4-diamine; TAPB: 1,3,5-tris(4-aminophenyl)benzene; TAM: 4,4′,4″,4‴-methanetetrayltetraaniline; BDA: Terephthalaldehyde; DFTA: 2,5-dihydroxyterephthalaldehyde; TFB: Benzene-1,3,5-tricarbaldehyde; BPDA: [1,1′-biphenyl]-4,4′-dicarbaldehyde; Sa: 2-hydroxybenzene-1,3,5-tricarbaldehyde; TFP: 2,4,6-trihydroxybenzene-1,3,5-tricarbaldehyde.

Membrane-based separations primarily utilize the sieving mechanism to eliminate organic and inorganic impurities. The pore size distribution of a membrane acts as a physical barrier that selectively allows small molecules (e.g., water) to pass and blocks large ones.^4^ As a result, separation performance is directly influenced by the average pore size of a COF membrane, which is determined by the pore sizes of the respective COF structures. In general, the pore size for RO and NF membranes range from 0.1 to 0.5 nm and 0.5 to 2 nm, respectively. Since uniform pore sizes with narrow distributions are desirable in a membrane, the symmetric precursor geometries and periodic topological properties of COF materials could result in highly uniform and ordered pores, enabling them to be excellent candidates for membrane fabrication.^7,11^ For example, among the different COF topologies (Figure 1a), the co-condensation reaction between the linear and trigonal linkers with triangular symmetry results in the formation of sheets with hexagonal pores, which subsequently form a framework (via π–π interaction) consisting of one-dimensional (1D) channels. According to an excellent review article^7^ that critically discusses the structural and functional traits of COFs, the intrinsic pore size of the typical COFs ranges between 1.1 and 5.3 nm. By manipulating linker geometries and using postsynthetic functionalization techniques, it is possible to achieve COFs with pore sizes less than 1 nm (i.e., 0.65 to 0.9 nm).^21,43^ This information, in fact, holds a significant value, particularly when it comes to the expectation of water purification using COF membranes. Apparently, based on the intrinsic pore size range, the COF membranes should not be expected to perform seawater desalination without further reduction in their pore sizes.

**Figure 2:**
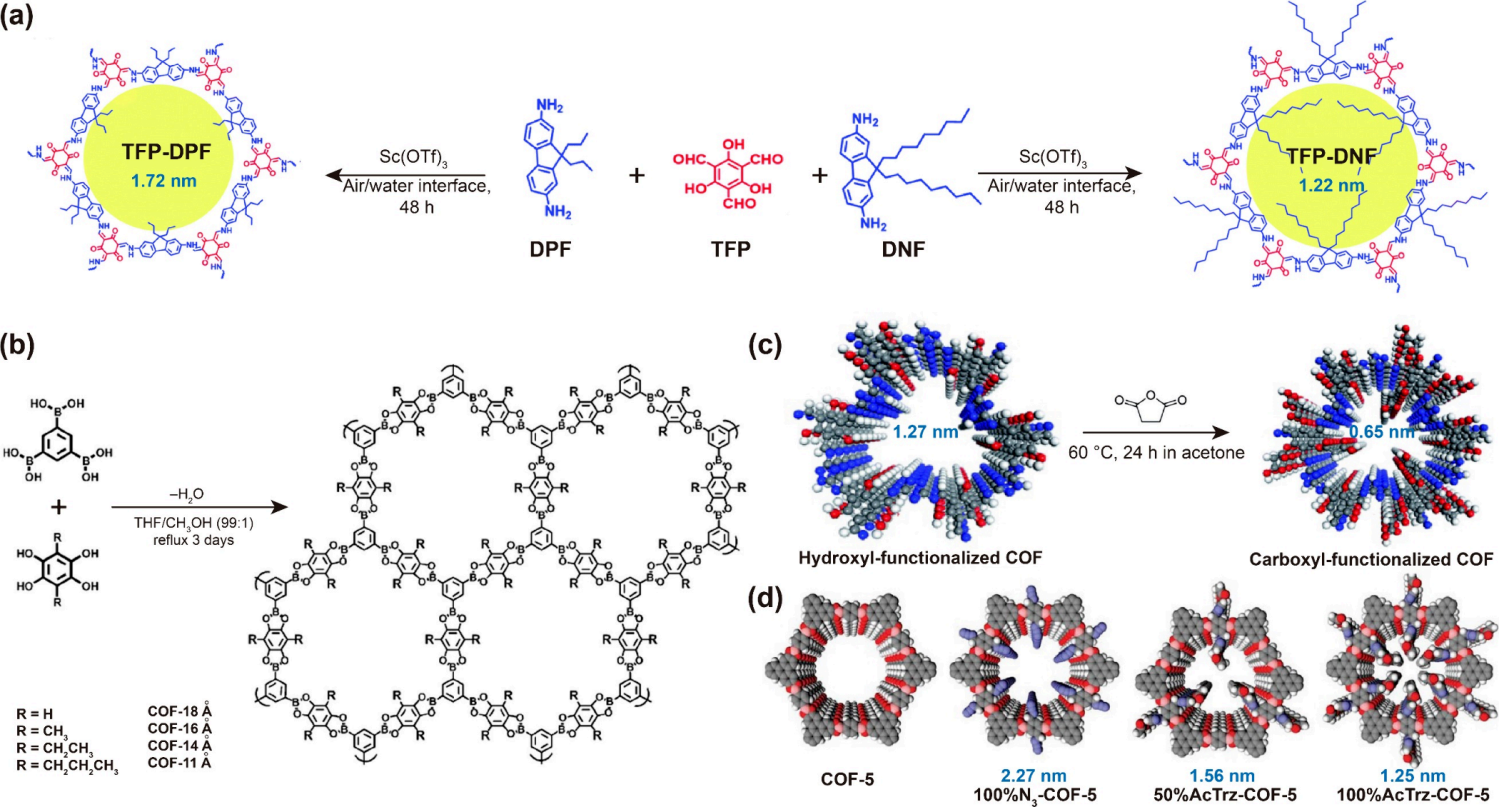
Pore engineering strategies for COF materials and membranes. (a–b) Examples of bottom-up approaches involving the use of monomers/precursors with different carbon chains; and (c) and (d) postsynthesis functionalization with carboxyl, acetyl, or ethynyl groups. Reproduced with permission from ref (47) Copyright 2019 Royal Society of Chemistry; ref (46) Copyright 2008 Wiley-VCH; ref (21) Copyright 2019 Royal Society of Chemistry; ref (48) Copyright 2011 Springer Nature.

The pore size of COFs could be tuned or adjusted by manipulating the precursor monomers (e.g., aldehydes and amines, Figure 1b–e) as well as modifying or functionalizing COFs before or after synthesis.^7,11,22,44,45^ For instance, Furukawa and Yaghi developed a series of COFs, including COF-1 (pore size = 0.9 nm), COF-5 (pore size = 2.7 nm), COF-6 (pore size = 0.9 nm), COF-8 (pore size = 1.6 nm), and COF-10 (pore size = 3.2 nm). While COF-1 was synthesized by the self-condensation reaction of 1,4-benzenediboronic acid (BDBA), co-condensation reactions of 2,3,6,7,10,11-hexahydroxytriphenylene (HHTP) with linear and triangular boronic acid based linkers were performed to prepare COF-5, COF-6, COF-8, and COF-10. This indicated that the pore size of COFs could be tuned simply by changing linkers’ geometries.^43^ Elsewhere, a three- [1 Knot + 2 Linkers] or four-component [1 Knot + 3 Linkers] mix linkers strategy was developed for the design and synthesis of crystalline COFs. Compared to conventional [1 Knot + 1 Linker] approach, the multicomponent system allows the synthesis of tailor-made COF structures with specially shaped pores, narrow pore size distribution, and enhanced structural complexity.^44^ Free-standing COF membranes were prepared via the interfacial polymerization (IP) reaction of the aldehyde monomer 1,3,5-triformylphloroglucinol (Tp) with 2,2′-bipyridine-5,5′-diamine (Bpy), 4,4′-azodianiline (Azo), 4,4′,4″-(1,3,5-triazine-2,4,6-triyl) tris(1,1′-biphenyl) trianiline (Ttba), and 4,4′,4″-(1,3,5-triazine-2,4,6-triyl) trianiline (Tta). According to the nonlocal density functional theory (NLDFT) calculations, the peak pore size of the free-standing COF membrane was 1.4 nm for the Tp-Tta, 1.9 nm for Tp-Ttba, 2.5 nm for Tp-Bpy, and 2.6 nm for Tp-Azo membrane.^10^ The strategies for tuning the pore size has also been executed by employing different carbon chains (Figure 2). In another study, after the reaction of 1,3,5-benzenetriboronic acid with 2,6-dialkyl substituted derivatives of 1,2,4,5-tetrahydroxybenzene (where R = H, CH_3_, CH_2_CH_3_, and CH_2_CH_2_CH_3_), the peak pore size of the functionalized COF powder was found to be correlated with the length of the carbon chains in the substituted derivatives (Figure 2b).^46^ Similarly, Shinde et al. developed two free-standing membranes by Schiff-base condensation reactions of Tp with 9,9-dinonylfluorene-2,7-diamine (DNF), and 9,9-dipropylfluorene-2,7-diamine (DPF). Owing to the longer carbon chains in the DNF monomer, the peak pore size of Tp-DNF membrane was smaller (1.22 nm) as compared to that (1.72 nm) obtained for Tp-DPF membrane (Figure 2a).^47^ The postsynthetic strategies to tune the pore size of COFs generally include their functionalization using carboxylic groups and click reactions involving acetyl and ethynyl groups.^20,21,48^ Although these functionalization strategies are effective, the pore sizes of the COF materials or membranes have been observed to remain above 1 nm. This indicates that the pore sizes of most COF materials and membranes are significantly higher than the commercial polyamide NF90 and NF270 membranes, which have a pore size range of 0.58–0.68 nm and 0.71–0.84 nm, respectively.^49,50^

### 2.2 Engineering the Surface Charge and Hydrophilicity

Nonsieving mechanisms, including electrostatic repulsion and Donnan exclusion, contribute to the separation of charged impurities by NF and RO.^4,51^ The surface charge of the COFs could be enhanced or regulated by selecting from a range of quaternary amine monomers (e.g., ethidium bromide) or by grafting the COF membrane with different functional groups such as −COOH, −OH, −NH_2_, and −SO_3_H.^52−56^ The resulting charged COF membranes have predominantly been studied for proton conduction and as an ion-exchange membrane.^54,56−58^

According to a recent study, an anionic COF membrane was prepared via a condensation reaction (water/oil interface) of Tp with *p*-toluenesulfonic acid (PTSA) and sodium 2,5-diaminobenzenesulfonate (Pa-SO_3_Na). Owing to its highly negative surface charge (−58.02 mV) induced by −SO_3_ groups, the TpPa-SO_3_Na membrane was observed to achieve efficient rejection (>99%) of cationic dyes as a result of electrostatic repulsion.^53^ In another study, researchers observed that by incorporating negatively charged −COOH groups, the functionalization of a COF membrane led to a significant reduction in pore size, from 1.27 to 0.65 nm.^21^ The reduced pore size and highly negatively charged surface of −COOH functionalized COF membrane was claimed to improve rejection of Na_2_SO_4_, NaCl, MgSO_4_, MgCl_2_, and FeCl_3_ by approximately 23%, 26%, 21%, 25%, and 10%, respectively.^21^ The −COOH functionalized COF membrane still could not outperform the commercial NF270 and NF290 membranes that are negatively charged (around −25 to −30 mV). This is because their mean pore size (0.58–0.68 nm and 0.71–0.84 nm, respectively) is slightly lower than that obtained for −COOH functionalized COF membrane (0.65–1.60 nm).^22,49,59^ It is well-documented that the size of the largest pore governs the separation performance and selectivity of a membrane.^60^ The studies on COF membranes generally articulate discussion revolving around performance and selectivity based on the intrinsic pore size or peak pore size, whereas the largest pore size is often ignored.^12^ Additionally, the rejection performance is attributed to electrostatic interaction or Donnan repulsion without actually measuring the surface charge or effect of applied pressure.^10,21^ These aspects must be taken into consideration in future studies on COF membranes to explain the performance and should be discussed to provide a complete picture and their applicability in the water industry.

**Figure 3:**
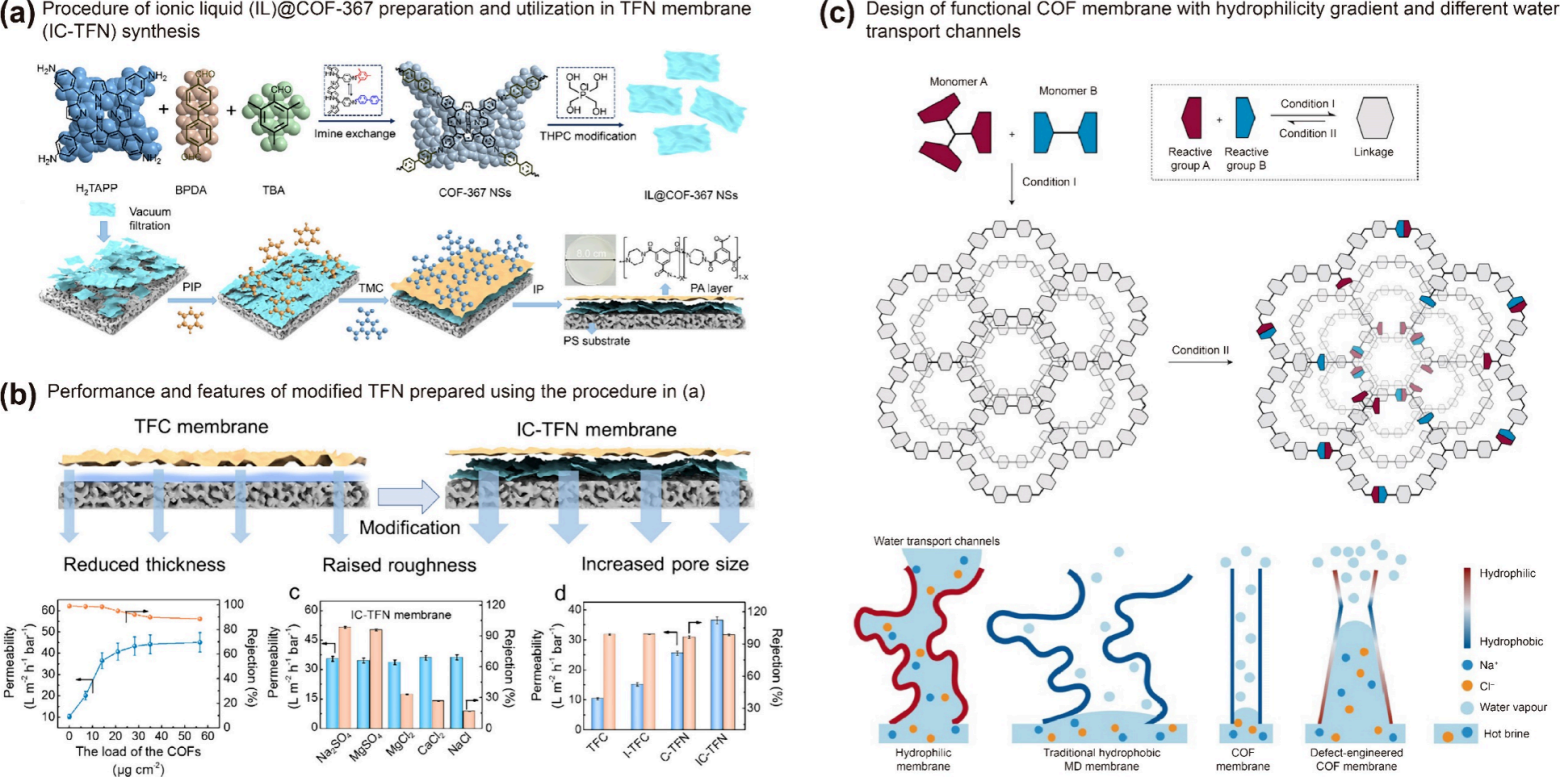
(a) Modification of thin film nanocomposite (TFN) membranes using ionic liquid modified COF-367. (b) Performance of conventional TFN, ionic liquid (I)-TFN, COF-367 (C)-TFN, and IC-TFN membranes in terms of water permeability and salt rejection. (c) Design of COF membrane with hydrophilicity gradient for MD. Reproduced with permission from ref (69) Copyright 2022 Elsevier; and ref (40) Copyright 2021 Springer Nature.

Depending on the operation type (e.g., pressure-driven or temperature gradient-driven), the hydrophilicity of COFs should be considered and adjusted. Hydrophilicity (contact angle, Θ < 50°) is a desired trait in pressure-driven systems such as NF and RO, while hydrophobic membranes are required for MD.^49,61,62^ Because COFs possess high surface areas (up to 2300 m^2^ g^–1^) and are hydrophobic, they were initially assessed as adsorbents in environmental remediation of toxic metals and organic micropollutants.^7,8,63^ Therefore, for the purpose of applications in aqueous media, hydrophilic COF membranes have been prepared either by selecting monomers containing hydrophilic functional groups (imine or β-ketoenamine) or by functionalizing the as-prepared COF membrane with hydrophilic groups such as −OH, and −COOH.^24,64−68^ Accordingly, the water flux of hydrophilic COF membranes has been observed to improve with antifouling properties. In a recent study, surface modification of commercial polyvinylidene fluoride (PVDF) UF membrane with a COF made from benzidine and 1,3,5-triformylbenzene (TzTb), and a poly(acrylic acid) resulted in a highly hydrophilic membrane (from Θ = 77° to Θ = 50°), improving the water flux by 1.82 times without compromising the rejection of model foulants including sodium alginate (SA), and bovine serum albumin (BSA).^37^ Another strategy could be to use hydrophilic COF nanosheets as interlayers or substrates for the fabrication of conventional TFN membranes (Figure 3a–b). This endowed highly hydrophilic behavior to the TFN membrane, resulting in improved water permeability (3-fold) without affecting the extent of salt rejection.^69^

In the past five years, MD technology has gained significant momentum for seawater desalination and hyper-saline water treatment. It is essentially a thermal process with a temperature gradient across the membrane as the driving force for water transport in vapor form, with drawbacks of low water flux, high pore-wetting, and high membrane scaling.^70,71^ In an effort to address the shortcomings, Zhao et al. reported an innovative approach to create a hydrophilic gradient in a COF-based MD membrane by selectively eliminating imine bonds, starting from the surface and moving toward the interior (Figure 3c). This strategy resulted in a significant increase in water vapor permeability, with a flux (600 L m^–2^ h^–1^) nearly three times higher than a commercial MD membrane.^40^ Since this is the only example of a COF-based MD membrane in the available literature, it is expected that future studies on this topic will provide further insights into separation and water transport mechanisms, pore-wetting issues, and the antiscaling behavior of the COF-based MD membranes.

As discussed above, pore size and hydrophilicity of COF membranes can be adjusted by introducing polar functional groups (particularly carboxylic acids, −COOH), which have shown significant improvements in water flux and pollutant rejection efficiency.^21,24,64−68^ The reported benefits of −COOH@COF membranes include enhanced hydrophilicity, reduced pore size, enhanced negative surface charge, improved salt rejection, and antifouling behavior.^21,24^ However, the relevant literature, to the best of our knowledge, reported separation performances based on short-term treatments using a single salt (excluding Ca^2+^) or model organic foulant, which does not reveal the full extent of introducing functional groups. It is known that during the synthesis of commercial polyamide NF, and RO membranes, hydrolysis of acyl chloride groups result in the formation of −COOH groups that are undesirable due to their critical role in membrane fouling. Since inorganic (Na^+^, Mg^2+^, and Ca^2+^) and organic (proteins and humic substances) impurities coexist in water, wastewater, and seawater, the inorganic impurities, especially Ca^2+^ even at a very low concentration, coordinate with the −COOH groups to form a scaling layer. This subsequently interacts with organic impurities for organic–inorganic complex formation. This interaction of −COOH groups with organics and inorganics instigate rapid membrane fouling, necessitating frequent membrane cleaning.^72−74^ Therefore, reducing the density of −COOH or shielding of −COOH groups on the membrane surface has been advised for antifouling and antiscaling behavior.^72,75^ Research on COF membranes should take note of the technological and process challenges associated with commercial membranes and devise suitable strategies accordingly to fast-track the practical application of COF membranes.

### 2.3 Stability of COF Membranes in Aqueous Media

The thermal and chemical stability of the membranes is crucial to ensure the long-term operation of the COF membranes. According to the available literature, various types of COFs such as COF-1, COF-5, COF-42, COF-119, and COF-701 have been reported to show good chemical (NaOH or HCl) or thermal stability (280–600 °C).^41^ However, the stability of COF membranes in water and chlorine is critical for applications in the water industry. Despite the significant research output, a major flaw of the membranes from two-dimensional materials, such as graphene, graphene oxide, and MXene, is due to their poor water stability, which results in membrane swelling or disintegration that leads to the expansion in pore size and reduction in pollutant rejection efficiency.^4^ The initial linkages exploited in the COF synthesis include boroxines (COF-1) and boronate esters (COF-5), which are prone to nucleophilic attack and disintegration.^9,76^ This results in the decomposition of COF-1 and COF-5 following prolonged exposure to water, limiting their application in water treatment. On the other hand, COF stability in water could be improved using triazine-, hydrazone-, and azine-linked COFs due to increased stability of chemical bonds in their structures, providing structural integrity against prolonged exposure to water.^9,76,77^ Other strategies to improve the water stability of COFs include the introduction of hydrogen bonding and linkage conversion, for example, imine to amide linkage.^78−80^ The stability of COF membranes in water is generally assessed by immersing them in water and is observed visually and using X-ray diffraction analysis (XRD) to see the disintegration of COFs. This may not provide an accurate depiction unless the cross-section of COF membrane is investigated for swelling, and the pollutant rejection performance is studied. To the best of our knowledge, the stability of COF membranes against the commonly used membrane cleaning agent sodium hypochlorite (NaOCl) has yet to be explored. Depending on the solution pH, dissociated species of NaOCl, including HOCl, OCl and Cl_2_ could degrade the commercial polyamide TFN membranes *via N*-chlorination, C–N bond cleavage, and hydrolysis. These effects ultimately inhibit the efficacy of membranes (water flux and salt rejection) and reduce the lifespan of the membranes.^81,82^ Therefore, without proven stability against chlorination, the practical application of COF membranes in water treatment will be difficult.

## 3 Evaluating the Processability of COFs into Membranes

COFs are challenging to process due to their intrinsic covalent backbone and rigid framework, which limits their solubility in aqueous media and common organic solvents. Without postsynthetic modification, COFs may not be made processable for real world applications like other polymers. Initially, studies involved conventional fabrication methods, such as spin-coating and drop-casting methods, but did not yield much success,^83,84^ which was again attributed to the poor processability of COFs. In arguably the first successful attempt, Colson et al. developed a COF-based thin film using an *in situ* growth method, whereby COF-5 film was grown over a monolayer of graphene.^85^ Notably, the standalone COF membranes for water treatment applications came much later, in 2017.^10,11^ The COF membrane fabrication methods have already been reviewed by Zhang et al.^42^ and Wang et al.^41^ and could be consulted for comprehensive details of the investigated strategies. In addition, progress in the strategies to tune or adjust the intrinsic properties of COFs have already been discussed in Section 2. In this section, we mainly focus on the challenges associated with COF membrane fabrication methods and solutions to address these issues.

### 3.1 *In Situ* Growth and Interfacial Polymerization Methods

**Figure 4:**
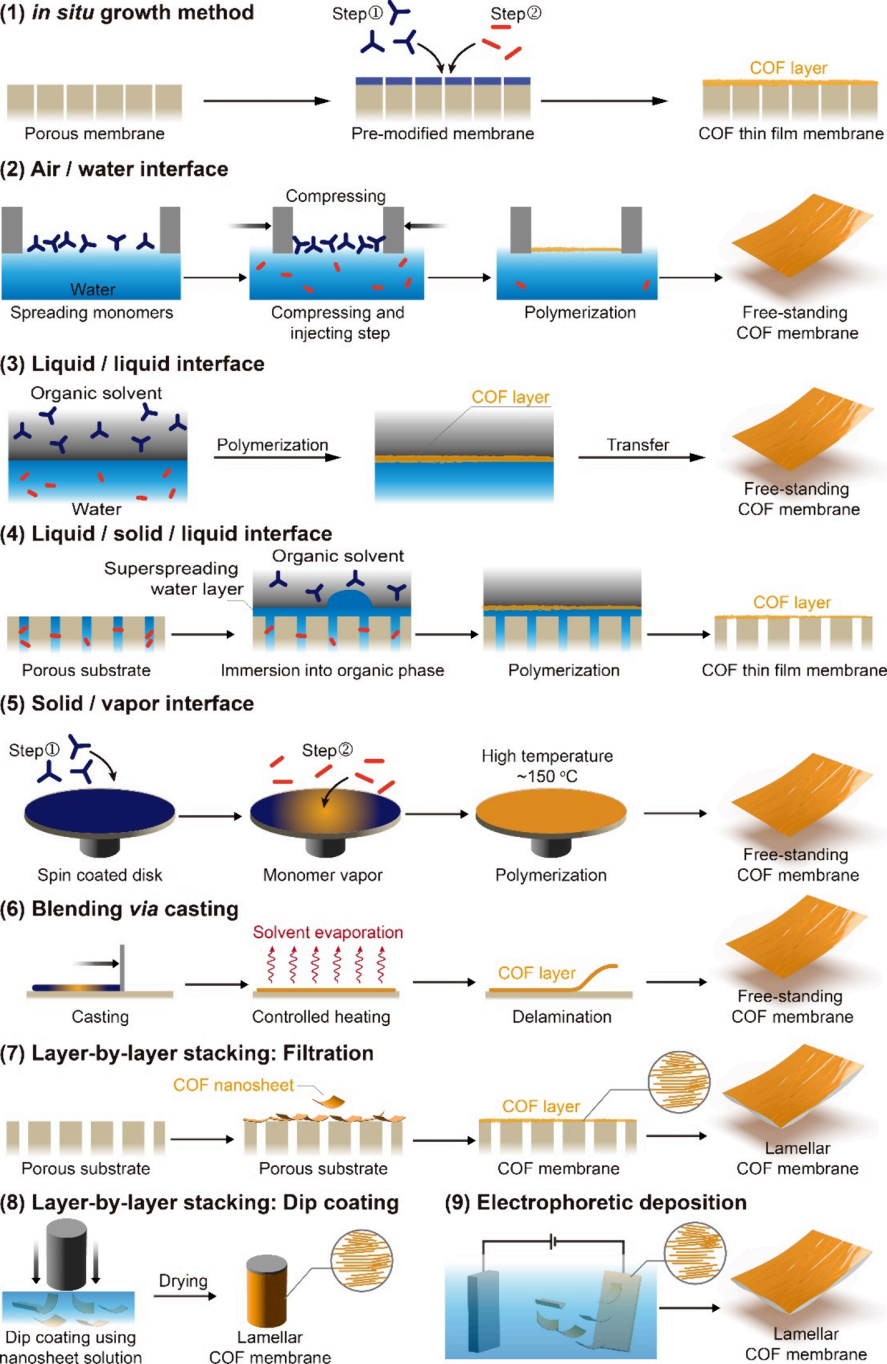
Methods for the fabrication of COF membranes reported in the literature.

For a feasible application in water treatment, it is ideal if the building blocks of COF membranes were stable in aqueous media, and should be able to attain crystallinity under ambient or mild conditions. In this context, the COFs synthesized without requiring deoxygenation or degassing are most suitable. COF films could be directly grown onto a porous substrate using *in situ* growth methods (Figure 4). On the other hand, IP methods involving C–N linkages between an aldehyde and an amine monomer (Figure 1d) have unsurprisingly become popular for the fabrication of COF membranes due to ease and mild reaction conditions.^10,86−88^ This is also because COFs are often synthesized as a fluffy crystalline powder with poor solubility, and their transformation into a thin-film best occurs at the interface by suppressing secondary monomer(s) reactions in the homogeneous phase. The secondary reactions, then, could inhibit the formation of defect-free membranes and can consume the monomers.

With regards to the *in situ* growth method, the formation of uniform, defect-free, ultrathin, and crystalline membrane on a porous substrate with narrow pore size distribution has not yet been realized in membrane technology.^5,89,90^ It is expected that ultrathin (subμm) polycrystalline membranes may not be possible because nucleation into large crystals is thermodynamically favorable, as per Ostwald ripening and Wulff construction theory. Therefore, a polycrystalline membrane (e.g., MOF membranes) containing even a single layer of crystals could be as thick as 1 μm.^91^ On the other hand, in the case of COFs, reduction in the surface energy of crystals due to Ostwald ripening may facilitate the development of ultrathin COF membranes. Indeed, owing to Ostwald ripening, the rod-shaped morphology of COF crystals progressed into thin (20–40 nm) hollow spherical crystals over time.^92^ Therefore, the fabrication of COF membranes with subμm thickness *via in situ* growth has been considered less challenging as compared to the MOF membranes.^12,91,93,94^ Another challenge during the *in situ* growth method of COF membrane is the uniform assembly of COF film on porous substrate, which requires that the COF layer should be thick enough to cover all the pores of the porous substrate to avoid defect creation. This issue could be solved by either functionalizing the porous substrate using nanosheets, nanoparticles, or functional groups.^12^

**Figure 5:**
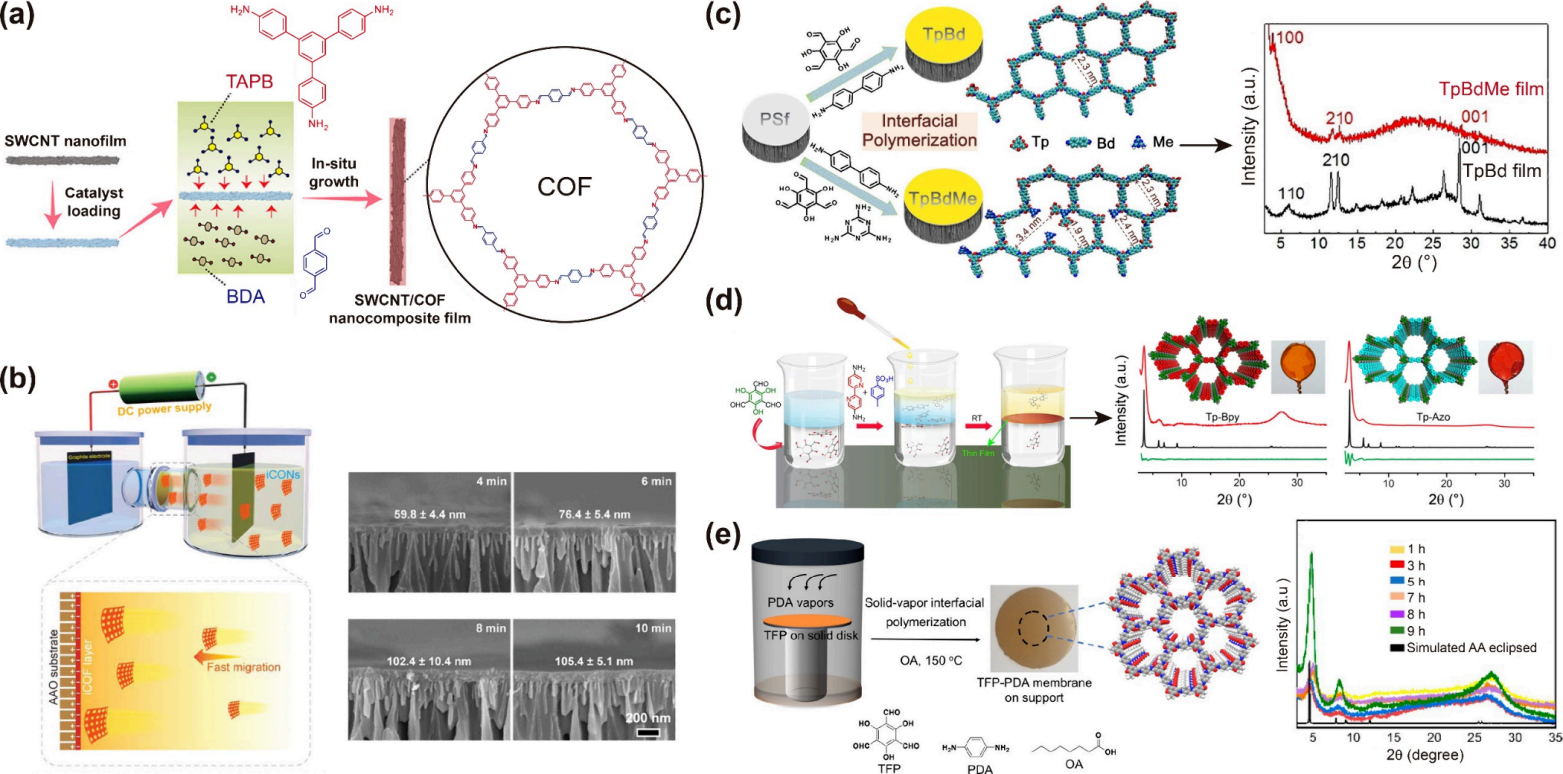
Strategies employed in the literature for rapid fabrication of COF membranes: (a) Contra-diffusion method for substrate-assisted interfacial polymerization (IP); and (b) Electrophoretic deposition method to assemble ionic (i)-COF nanosheets and thickness of COF membrane as a function of electrophoretic deposition time. Examples showing the methods for fabricating COF membranes with and without excellent crystallinity: (c) *in situ* method of COF membrane fabrication on a porous Polysulfone (PSf) membrane with poor crystallinity; (d) Role of a buffer layer in IP method on the crystallinity of COF membranes; and (e) solid–vapor IP method for obtaining highly crystalline COF membranes. Reproduced with permission from ref (13) Copyright 2022 Wiley-VCH; ref (93) Copyright 2022 American Chemical Society; ref (29) Copyright 2019 Elsevier; ref (10) Copyright 2017 American Chemical Society; and ref (88) Copyright 2020 American Chemical Society.

In addition to the ultrathin membranes being a prerequisite, rapid synthesis of COF membrane is another area that requires attention. Currently, the overall time to fabricate COF membranes has been reported to range between a few hours to a few days, which is significantly longer than the time (up to 1 h) required for the fabrication of conventional TFN membranes.^10,25,95,96^ Nevertheless, Monoranjan et al. developed a simple contra-diffusion procedure to synthesize a COF membrane with 58 nm thickness in 10 min by executing the IP reaction between TAPB and BDA on a porous substrate that is modified using single-walled carbon nanotubes and polydopamine (Figure 5a).^93^ Similarly, in a recent study, Wang et al. reported a rapid strategy to fabricate a COF membrane using an electrophoretic deposition method, whereby ionic COF nanosheets could be assembled into a membrane in just 6 min.^13^ This method was claimed to be not only scalable but could also provide effective control over the thickness of the COF membrane by merely changing the electrophoretic deposition time (Figure 5b). However, it is to be noted that the synthesis and exfoliation of ultrathin 2D COF nanosheets without losing structural integrity remain a considerable challenge.

The crystallinity of polymers in polymeric membranes governs the channel or pore structure and is essential to attain high selectivity and permeability. COF powders exhibit relatively better crystallinity than COF membranes, which is influenced by their intrinsic properties, rate of reaction during membrane fabrication, and flexibility. It is convenient to achieve high crystallinity in frameworks resulting from reversible linkages (i.e., can break easily) as they can correct the error to ensure a highly crystalline product. This is one of the reasons that hydrogen-bonded organic frameworks are easy to crystallize due to the low dissociation energies of their hydrogen bonds.^6^ On the other hand, COFs consist of strong covalent bonds, which may require elevated temperatures to ensure the conversion of amorphous to stable crystalline products. The IP method is usually carried out under ambient conditions, and may not yield highly crystalline COF membranes. Thus, they may not be applied to most COF-based membrane production due to the poor solubility of monomers and harsh reaction conditions. Furthermore, the poor stability of amine monomers at high temperatures is a hurdle in the high temperature synthesis of COF membranes.^95,97,98^

The crystallinity of COF membranes can be improved by (i) providing a buffer layer between the aqueous and organic interface (Figure 5d);^10^ (ii) limiting the diffusion speed of the monomers toward the reaction zone;^99^ (iii) employing a different interface such as solid–vapor at high temperatures (around 150 °C, Figure 5e);^88^ and (iv) selecting suitable monomers (e.g., tetrafluorophthalonitrile) that can form rigid bonds, thereby promoting the formation of ordered crystalline structures.^100,101^

In summary, to obtain a crystalline COF membrane, the fabrication process can benefit from high temperature and limited monomer diffusion. However, no strict guidelines, criteria, or predictive models could be universally followed for COF membrane fabrication. Crystallinity of COF membranes are commonly evaluated using X-ray diffraction (XRD) and high-resolution transmission electron microscopy (HR-TEM). The XRD peaks (along with the theoretical simulations) represent crystallinity in COF membranes, and were observed to be less intense or broad depending on the grain size or amorphous content.^11,27,88,102,103^ On the other hand, it is now an accepted norm to show HR-TEM images of selected areas to claim crystallinity in COF membranes.^102,104^ This implies that the majority of the COF-based membranes reported in the literature could be classified as polycrystalline membranes. In the last six years, different fabrication strategies, including functionalization approaches, have been developed as new contributions to the field of membranes. At the same time, the process parameters related to water treatment, such as removal and transport mechanisms as well as process optimization, and cost-benefit analysis, have been largely overlooked. These shortcomings have been explained further and discussed in Section 4 below.

### 3.2 Blending or Incorporation in Conventional Thin Film Nanocomposites

**Figure 6:**
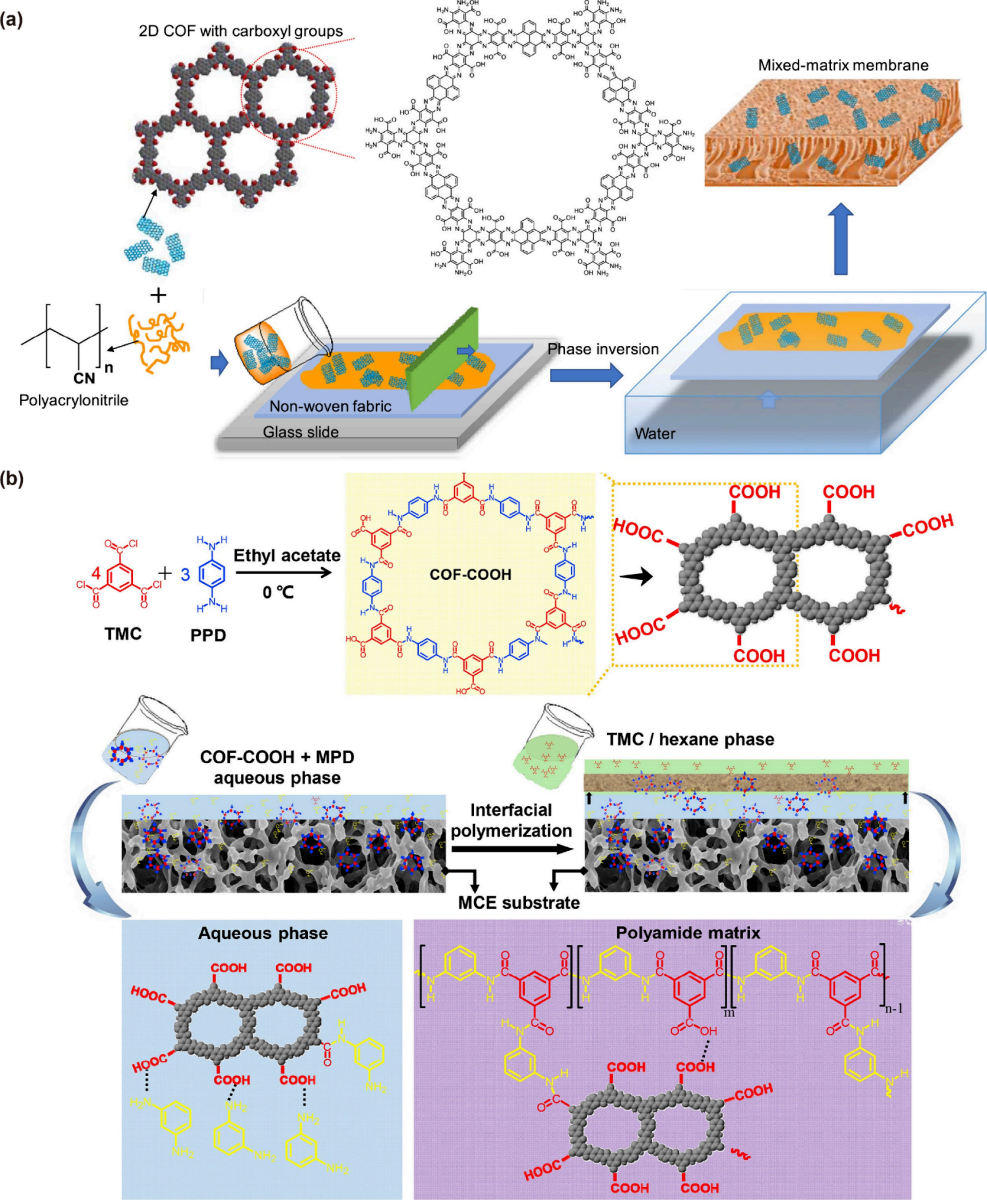
(a) Functionalized COF-embedded PAN MMMs prepared using a nonsolvent induced phase inversion method. (b) Incorporation of functionalized COF in fabrication of polyamide FO membrane using IP method. Reproduced with permission from ref (24) Copyright 2019 Elsevier; and ref (39) Copyright 2020 Elsevier.

Through conventional methods of IP and *in situ* growth, COF membranes with subnm pore sizes could not be realized, which limits their application in water treatment industry, especially desalination.^10,29,88,105^ As explained earlier in Section 2.1, the pore size of a COF membrane could be tuned to some extent by functionalization. Pore size distribution can also be narrowed or tuned by blending COFs into other matrices (Figure 6a) or by their incorporation in TFN membrane fabrication (Figure 6b).^15,69^ With a few exceptions, the COF-embedded mixed matrix membranes (MMMs) are not successful in developing COF membranes with subnm pores and therefore, predominantly used for UF applications. For instance, Xu et al. developed the first COF-MMMs using a nonsolvent induced phase inversion method by blending COF prepared using Tp and 2,5-imethyl-1,4-phenylenediamine with polysulfone (PSf). The COF-MMM demonstrated enhanced hydrophilicity and antifouling behavior as well as better permeate flux and rejection of a model foulant (humic acid).^14^ Similarly, when −COOH functionalized COF (0.8% w/w) was used as a nanofiller in a polyacrylonitrile (PAN) membrane, the surface of the MMM became almost completely resistant to organic foulants such as bovine serum albumin (BSA).^24^ In a recent study, Xu et al. demonstrated that embedding of 1,3,5-triformylbenzene (Tb)-benzidine (Bd) COF in a PVDF membrane is beneficial for the removal of lead (>85%) via chemisorption.^36^

COF-MMMs have also been applied in desalination.^15,38^ The first strategy was to synthesize nanocomposite-containing COF nanosheets and 1D cellulose nanofibers, followed by vacuum-assisted filtration on a porous substrate. The MMM membrane obtained using this method had a pore size range of 0.45–1.00 nm and could maintain Na_2_SO_4_ rejection of 97% at 43 L m^–2^ h^–1^ bar^–1^.^15^ The second strategy was to incorporate sulfonated COFs in cellulose triacetate/cellulose acetate matrix to obtain an FO membrane with improved physicochemical properties, which surpassed the commercial membrane in terms of permeate flux and selectivity by 2–3 times.^38^ Despite the reported enhancements in performance, we believe that the dispersibility of COFs in solvents still needs to be improved for blending into different polymeric matrix, providing better control over the structural features of MMMs.

Wang et al. incorporated an amine-rich Schiff base networks (SNW-1) COF as a filler by dissolving it in water containing piperazine with the hypothesis that the −NH groups of SNW-1 COF would make strong covalent bonds with the −C(=O)Cl groups of trimesoyl chloride (TMC). This rendered enhanced stability to the modified polyamide membrane. However, this strategy was only successful in achieving higher permeate flux (almost 2-fold) at a slightly reduced salt rejection as compared to an unmodified polyamide membrane.^16^ In another study, COOH–COF were synthesized and added to the aqueous phase containing *m*-phenylenediamine (MPD) that reacted with TMC to form an FO TFN membrane with better hydrophilicity and narrower pore size distribution. This, in turn, facilitated in achieving higher permeate flux (4-fold) and reduced reverse salt flux than the unmodified TFN membrane.^39^ Other studies have also reported the incorporation of COF particles,^106^ COF nanosheets,^18,69^ and COF thin-films^35,107^ as fillers, scaffolds, or interlayers to improve the performance, i.e., permselectivity. Although the incorporation of a COF into a polyamide TFN membrane generated exceptional results in terms of desalination performance and permeate flux, the contributions of COFs in pore size distribution and removal mechanisms remain unclear and unexplored. Researchers will have to consider if the standalone COF membranes would be able to create their own individual identity among other polymeric membranes in water treatment or if COFs are better applied as fillers or binding materials.

The majority of the membrane preparation methods discussed above are based on 2D COFs. The development of membranes using 3D COFs could be attractive for applications in water treatment because of their abundant open water transport channels, noticeably reduced pore size, large surface areas, and promising stability, as compared to 2D COFs.^108,109^ However, literature on such application of 3D COF-based membranes is limited due to difficulty in their synthesis. Notably, since anisotropism is not apparent, controlling the thickness of 3D COF-based membranes is challenging. The current examples of 3D COF-based membranes are derived from COF-300^110,111^ and SNW-1.^66^ Niu et al.^110^ prepared MMMs by casting the mixture of imine-linked COF-300 into polystyrene for lithium separation. In another study, *in situ* growth of COF-300 was performed on an NH_2_-modified ceramic support using solvothermal method.^111^ The COF-300 had a pore size ranging from 0.89 to 1.68 nm, and the tabular COF-300 membrane achieved >99% removal of chrome black T dye at 80 L m^–2^ h^–1^ bar^–1^.^111^ Yang et al.^66^ developed a composite membrane by coating SNW-1 COF on PAN substrate for a pervaporation application. The peak pore size of 3D COFs is smaller than 2D COFs,^7,108,109^ but it should be of note that their pore size may not accurately represent the pore size of the 2D/3D COF derived membranes.

### 3.3 Electrospinning and 3D Printing Methods

In addition to the in situ growth, interfacial polymerization, and blending methods (Figure 4), two emerging techniques, namely electrospinning^112−114^ and 3D printing,^115^ have been investigated for the conversion of COF powders into membranes. Electrospun nanofiber technology involves spinning a casting solution under high voltage to prepare fibrous membranes. These membranes contain nanofibers with diameters ranging from 10 to 100 nm, demonstrating high porosity, large surface area, tunable pore size, and adjustable surface functionalities.^116,117^ Since COFs are not inherently soluble in common solvents, the preparation of pure electrospun COF-based nanofibrous membranes remains challenging. Nevertheless, Yan et al. successfully developed an electrospun nanofibrous membrane by suspending SNW-1 COF powders at different concentrations (5%, 10%, and 20%) in a PAN matrix.^112^ This SNW-1@PAN nanofibrous membrane was evaluated as a sorbent in pipet tip solid-phase extraction (SPE) for concentrating sulfonamide antibiotics before quantification using high-performance liquid chromatography (HPLC). Compared to other SPE sorbents, the SNW-1@PAN nanofibrous membrane exhibited excellent extraction efficiency, detection limit, and reusability.^112^ Similarly, in another study, Kang et al. prepared an electrospun nanofibrous membrane using a mixture of PAN and TFP-BD(NH_2_)_2_ COF,^113^ which was tested as an ion-exchange sorbent in pipet tip SPE for arsenic removal. The COF-incorporated PAN membrane demonstrated a selective sorption capacity of 33.9 μg/g for arsenic(V), along with excellent selectivity.^113^ While COF-suspended electrospun membranes have shown promising performance in solid-phase extraction for enrichment purposes, their potential in water treatment and selective adsorption for resource recovery remains to be explored.

Additive manufacturing or 3D printing is an emerging technology that enables rapid production of prototypes and proved to be quite useful in civil engineering, automotive, and aerospace. However, there is limited literature available on the preparation of COFs using 3D printing technology. In a first attempt, Zhang et al. introduced a method to incorporate COFs into 3D printed materials.^118^ They achieved this by coassembling a 3D printing template, Pluronic F127, with an amorphous imine polymer, resulting in printable hydrogels. After removing the F127 template and annealing the structure at temperatures ranging from 90 to 150 °C, 3D COF-based monoliths with high crystallinity, hierarchical pores, and mechanical stability were obtained.^118^ Then, Mohammed et al. developed a COF-based membrane using a 3D printed hydrogel consisting of graphene oxide (GO), imine-based COF, and water.^115^ The addition of GO in the blend facilitated the formation of distorted meso- and macro-pores for a COF-GO foam membrane. This unique structure enabled rapid sorption (within 30 s) of organic pollutants, such as bisphenol A, methylene blue, and basic fuschin.^115^ Incorporating COFs into 3D printing materials opens up new possibilities for the fabrication of functional structures with high crystallinity, hierarchical porosity, and mechanical stability. Further research and exploration of this area will contribute to advancing the morphology control of COF-based materials and their applications in water treatment.

## 4 Evaluating COF Membranes in Water Treatment

### 4.1 Permselectivity

**Figure 7:**
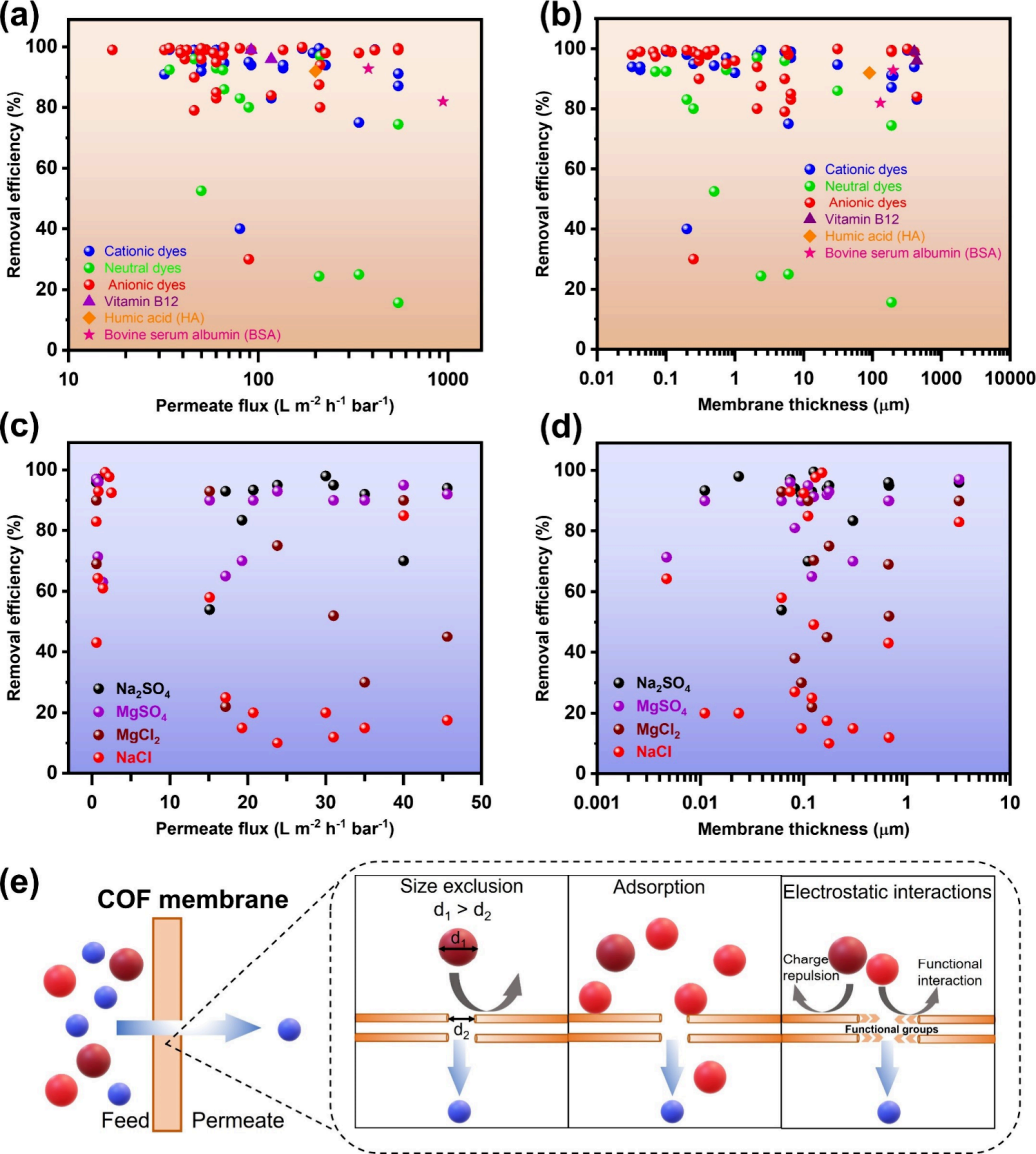
(a) Performance of COF-based membranes for the removal of organic pollutants (mainly organic dyes) as a function of permeate flux. (b) Performance of COF-based membranes for the removal of organic pollutants (mainly organic dyes) as a function of membrane thickness. (c) Desalination by COF-based membranes as a function of permeate flux. (d) Desalination by COF-based membrane as a function of membrane thickness. (e) Mechanisms of removal by COF membranes. Data plotted using the following sources: (a–b)^10,11,14,17,23−30,53,87,88,95,96,123−130^ and (c–d)^16,19,21,29,31−35,39,69,106,131−133^.

Covalent organic framework membranes are still in their infancy phase, and the focus of research has mainly been on the development of strategies for improved COF processability into a separation membrane. This is probably the reason that environmental engineering aspects have been largely ignored. Therefore, the breadth of water treatment technologies (such as RO, NF, or UF) in which COF membranes can be applied is relatively unknown. Although theoretical studies have shown great potential of triazine-COF membranes,^119^ −COOH/–NH_2_ functionalized COF membranes,^67^ and lamellar COF membranes^120^ for desalination, the experimental evidence suggested otherwise (Figure 7). The pore sizes of COF powders tend to range from 0.9 to 5.3 nm,^7^ and the resultant COF membranes (excluding COF-based composite membranes) have been reported to display a pore size of 0.3 nm^12^ to 3.2 nm.^43^ As expected, most COF membranes (pore size >1 nm) could be categorized as NF membranes and did not achieve effective desalination. This is because the hydrated ionic radii of undesired species present in seawater and brackish water, such as Na^+^ (0.358 nm), Ca^2+^ (0.412 nm), Mg^2+^ (0.428 nm), Cl^–^ (0.332 nm), and SO_4_^2–^ (0.379 nm), are significantly lower than the aperture of COF membranes.^121,122^ COF membranes prepared using IP or *in situ* growth methods have been predominantly investigated for the rejection of organic dyes (Figure 7a–b).^10,11,14,17,23−30,53,87,88,95,96,123−130^ According to this literature survey, COF membranes can achieve a median removal of 97% for cationic dyes, 83% for neutral dyes, and 98.6% for anionic dyes by the tested COF membranes, which could be attributed mainly to a size exclusion mechanism. In addition, we noted that the removal of anionic dyes is usually better than its counterpart, which could be credited to the electrostatic repulsion between anionic dyes and negatively charged COF membranes (Figure 7).

The separation of organic dyes by membranes is a worthy investigation considering their significant annual production (7 × 10^5^ tons/y) and applications in textile, cosmetics, and printing industries. In this domain, the performance of the COF membranes has been mostly studied under the following conditions: dye concentration = 2.5–100 mg L^–1^, applied pressure = 1–5 bar; and operating mode = dead-end filtration.^10,23,87^ Note that the dye-bath wastewater composition is complex and contains high concentrations of chemical oxygen demand (COD = 2700 mg L^–1^) and chloride (18000 mg L^–1^).^134^ Because of the simple operating protocols and highly practical solution chemistry that is already in use, the findings from these studies, notwithstanding the fabrication methods, have limited practical implications. Research on COF-NF membranes, therefore, needs to be refocused by developing solutions to the emerging challenges in the water industry.

Some of the outstanding synthesis-related issues of the NF membranes include the development of membranes with uniform or narrow pore size ranges, energy efficient membranes, fouling resistant membranes, and high-pressure membranes. On the process engineering side, it is suggested to apply COF-based NF membranes for advanced water treatment, such as secondary treated effluent and freshwater (surface and groundwater) for the removal of dissolved organic carbon, micropollutants (*e.g.,* pharmaceuticals), and heavy metals. The findings obtained from environmentally relevant water matrices would help in understanding the removal mechanisms and influencing factors, which could then be considered to fine-tune the properties of COF membranes.

Owing to their large pore size (above 0.5 nm), COF membranes may not be suitable for desalination. When the active layer of COFs is directly employed without the additives, the desalination performance of the COF membrane is generally poor. Nevertheless, COF functionalization^21^ or stacking into lamellar assembly^135^ or incorporation into conventional TFN membrane^16^ has been observed to obtain enhanced desalination performance, which is attributed to reduced pore size. Compared to the COF derived membranes used for dye separation, COF derived membranes assessed for desalination have two main differences: (i) relatively less thickness (mainly up to 1 μm) due to their incorporation into TFN membranes; and (ii) lower permeate flux due to smaller pore size (Figure 7c–d). Overall median removal by COF derived membranes is as follows: Na_2_SO_4_ (95%) > MgSO_4_ (90%) > MgCl_2_ (61%) > NaCl (46%).^16,19,21,29,31−35,39,69,106,131−133^ Notably, excellent removal (>90%) of NaCl has been achieved at the expanse of water permeability, i.e., less than 5 L m^–2^ h^–1^ bar^–1^.

A literature survey shows COF-based desalination membranes are mostly tested at a salt concentration of 1000–2000 mg L^–1^ and 5–10 bar pressure under dead-end filtration mode.^12,31,107,126^ First, these testing conditions should meet industry standards. For instance, the industry standards for testing under ambient conditions is as follows: (a) RO membrane (seawater): 32,000 mg L^–1^ of NaCl, 55.15 bar of hydraulic pressure, and cross-flow operating mode; (b) RO membrane (brackish water): 2,000 mg L^–1^ of NaCl, 15.5 bar of hydraulic pressure, and cross-flow operating mode; and (c) NF membrane: 2,000 mg L^–1^ of MgSO_4_, 4.8 bar of hydraulic pressure, cross-flow operating mode. Second, an important parameter (i.e., recovery rate) has not been considered or reported during the long-term operation of COF derived membranes in the literature. The effect of concentration polarization (accumulation of ions near the membrane surface) becomes significant with the increase in the recovery rate and may lead to rapid membrane fouling. This is because the sparingly soluble inorganic ions could form their respective hydroxides and precipitate on the membrane surface.^136^ Third, cross-flow filtration should be preferred over dead-end filtration because it allows continuous-flow operation and exerts higher shear stress to allow assessment of structural integrity. To the best of our knowledge, there is simply not enough evidence to claim exceptional permselectivity for COF-based membranes compared to other polymeric membranes and should not be highlighted excessively in research output.

Owing to their large surface area and high porosity, COFs have been investigated for removing or separating metals and radioactive elements.^137,138^ For example, Ma et al.^139^ developed COFs with carboxyl and triazine functionalities to achieve enhanced separation of lead (128 mg g^–1^) via a chemisorption mechanism. Xu et al.^36^ developed a composite UF membrane using a nonsolvent induced phase separation method by blending TbBd COF in PVDF. The composite COF/PVDF membrane achieved above 90% lead removal at a permeate flux of around 120 L m^–2^ h^–1^ bar^–1^ and maintained 87% removal after four cycles. However, the mechanisms of lead removal were not evaluated and explained.^36^ In another study,^140^ electrospun nanofibers using PAN/guanidinium-based ionic COFs were synthesized for efficient separation (173 mg g^–1^) of chromium(VI). These nanofibers were observed to remove chromium(VI) by ion exchange, hydrogen bonding, and electrostatic interaction mechanisms. Despite using the chemical desorption by 1 M NaOH and 0.1 M HCl, the PAN/COF nanofibers could not be completely regenerated, but it did not significantly affect the removal efficiency even after five cycles.^140^ Zhang et al. prepared a chitosan membrane loaded with hydrazone-linked COF (CM@COF) using freeze-casting method for the separation of several metals, including copper(II) and chromium(VI).^141^ The adsorption capacity of CM@COF was found to be 122 mg g^–1^ for copper and 388 mg g^–1^ for chromium.^141^ In other studies, tannic acid modified COF embedded FO membranes^142^ and interlaced-stacked COF/polysulfonamide NF membranes^143^ have been reported to achieve efficient removal (90–99%) of metals (such as lead, copper, and nickel) and rare-earth metals (such as lanthanum, neodymium, and yttrium), respectively. Wu et al. developed MMMs containing [NH_4_]^+^[COF–SO^3–^] and sulfonated-poly(ether sulfone) for the uranium extraction.^144^ The as-prepared MMMs achieved 99% uranium removal with an adsorption capacity of 99.4 mg g^–1^ (at pH = 1).^144^ In general, irrespective of COF type, incorporating a COF into a membrane matrix results in better removal of pollutants. The performance could be enhanced by tuning the properties of COFs before their incorporation into membranes to promote selective removal for resource recovery.

### 4.2 Separation Mechanisms

Similar to other membrane-based separation processes, mechanisms of removal by COF derived membranes include size exclusion, electrostatic interaction, functional group interaction, and adsorption (Figure 7e). In line with the available literature, size exclusion has been claimed as the dominant removal mechanism for the separation of organic impurities, followed by electrostatic repulsion.^11,23−30^ Size exclusion is generally claimed as a separation mechanism in COF derived membranes due to its inherently ordered pore structure consisting of linear water transport channels. It is to be noted that ordered periodicity could not be claimed for amorphous thin films, while the size exclusion mechanism is difficult to prove in the case of polycrystalline COF membranes containing both crystalline and amorphous regions and different grain boundaries.^10,31,87,88^ Because polycrystalline COF membranes are expected to be disoriented, the transport of molecules would be unpredictable and may follow a tortuous path similar to a nanoporous lamellar membrane.^4,135^ A recent study comprehensively showed that a thick polycrystalline COF pellet achieves excellent removal of organic dyes mainly via adsorption rather than a commonly claimed size exclusion mechanism. The operating conditions, such as flow rate and feed volume, could also be adjusted accordingly to maintain a high rejection efficiency.^145^ These findings are in line with the fact that COFs possess a high surface area—a desired trait in adsorbents—and have been employed for the adsorptive removal of organic and inorganic pollutants.^7^ Although it is hard to conclude that adsorption is the main removal mechanism in all COF derived membranes, future studies should focus on exploring the contribution of adsorption and size exclusion for the removal of organic dyes or shift their focus to use the adsorption capacity of COFs for selective removal of impurities such as heavy metals. We, once again, strongly suggest that using organic dyes as probe chemicals to assess separation performance may not be the best strategy.

With regards to the desalination, the pore sizes of COF derived membranes are to be reduced for achieving any appreciable salt rejection via size exclusion and electrostatic interactions.^19,31−35^ However, by doing so, the permeate flux of the COF derived membranes during desalination has been reported to be lower than those fabricated for organic dye removal (Figure 7). Compared to chloride salts, the rejection of sulfate salts was better. It could be attributed to the hydrated radii and valence ratio of ions.^21,32,69,133^ Indeed, based on the hydrated radii of ions and their valence ratio, salt rejection should be as follows: Na_2_SO_4_ (valence ratio = 2) > MgSO_4_ (valence ratio = 1) > MgCl_2_ (valence ratio = 0.5) > NaCl (valence ratio = 1). This implies that both size exclusion and electrostatic interactions contribute to salt rejection by COF derived membranes. Notably, according to the transition-state theory, these ions are required to cross an energy barrier, which is governed by the driving force (e.g., applied pressure), feed composition, degree of dehydration, and solute–membrane interactions. Ions can undergo dehydration under pressure, which reduces their size and subsequently reduces their rejection efficiency. However, these aspects remain unexplored and should be assessed to explain the selectivity of COF derived membranes for monovalent and divalent ions in single, binary, and complex systems to clarify the separation mechanisms further. This would also pave the way for the use of COF membranes to selectively separate and recover valuable metals (e.g., Li) from salt-lake brine. Finally, organic fouling of desalination membranes is a long-standing issue and may be investigated to showcase the efficacy of COF derived membranes.

## 5 Prospects and Challenges

COF derived membranes have made great strides in a little time span of about six years, especially in their syntheses. Initially, standalone pure COF membranes were the main focus, which then shifted to the *in situ* growth method, followed by COF functionalization and use of COFs as binders or interlayers in conventional TFNs.^10,22,69,96^ It appears that these shifts happened due to the poor crystallinity of the developed COF membranes or difficulty to attain a COF membrane with subnm pores. Otherwise, there is still a lot of room to develop a highly oriented crystalline COF membrane with ordered pores to ensure pore-based solute selectivity and transport. In addition, increasing the lateral size of the COF crystals may help avoid the impacts of defects on solute and solvent transport. Accordingly, the characterization of materials and the resultant membranes should be carefully executed to understand their physicochemical properties as well as transport behavior and separation mechanisms. For example, pore size distribution, membrane surface charge, and hydrophilicity could be analyzed using readily available equipment and provide a good indication of the hydraulic performance of a membrane.

Additionally, there is a need to standardize the procedure for measuring the pore size distribution. Pore size distributions of COF membranes have been estimated by the NLDFT model using N_2_ adsorption–desorption isotherms,^21^ a pore size analyzer,^14^ or the rejection of different molecules through calculating the Stokes radius.^12^ There might be slight variation in the estimated pore size distributions using these different methods. Therefore, we suggest that the largest pore size should be considered to explain the performance of COF membranes until evidence shows that only the peak pore size governs the solute separation and solvent transport behavior.

Modification of COFs and COF derived membranes has undoubtedly shown promise.^20,21,53^ The primary aim of these modifications is to reduce their pore size distributions. Notably, the functional groups within their pores can contribute to separation mechanisms by providing additional chemical interaction and binding sites for pollutants. However, the implication of these functionalities on structure and crystallinity, as well as permeate flux and fouling behavior, should be evaluated to make an informed decision on the suitability of certain functionalization. On the other hand, the intended purpose of COF incorporation into conventional TFN matrix is ambiguous and requires a clear pathway. COF addition for merely narrowing the pore size distribution and improving hydrophilicity of TFN membranes may not be cost-effective due to their costly synthesis, particularly when other cost-effective routes are available such as the fabrication of polyamide membranes using surfactant regulated IP processes.^90^ In addition to elucidating the role of COFs in COF-incorporated TFN membranes, understanding their effects on structural integrity and chemical stability (e.g., against chlorination) will be critical for their future contributions in applications. With the advances in COF derived membranes, a thorough understanding of their energy efficiency and environmental impacts will be required during process optimization and intensification.

Although still developing, the mechanical strength of free-standing COF membranes is not yet practical, and requires a support substrate to withstand the shear stress exerted during operations.^10^ COF membranes prepared by *in situ* growth on a substrate or using additives or templates improve their mechanical strength.^15,146^ Once pure COF membranes are ready for applications, different configurations, such as tubular and hollow fiber, could be developed for upscaling. Moreover, the integration of COF with other emerging materials can be investigated for enhancing performance and mechanical strength. For example, blending MOFs within COF derived membranes may improve the performance of MMMs, particularly the sorption capacity, due to the high surface area of MOFs. On the other hand, the incorporation of GO to COF derived membrane may endow the resultant MMMs with improved mechanical strength, enhanced hydrophilicity, and reduced swelling. These are desirable properties of a membrane in water treatment. All these aspects of COF derived MMMs need to be investigated to understand the extent of improvements.

Attaining high surface area and superhydrophobicity may not be challenging for COFs. Furthermore, COFs could easily be functionalized with both hydrophilic and hydrophobic groups. Therefore, the focus might benefit a shift from pressure-driven membranes to other membrane separation processes. For example, MD is an emerging desalination process that does not have a strict requirement of pore size range and can be operated using a microporous membrane.^71,73^ In the first and only study, a COF-based MD membrane outperformed the commercial MD membrane and maintained 99.99% rejection of NaCl at 370 L m^–2^ h^–1^ for 100 h at the feed NaCl concentration of 3.5% (w/w) and feed temperature of 75 °C.^40^ We expect that more research on COF-based MD membranes will follow and will assess the scaling issue caused by multivalent cations and mass transfer and temperature polarization. Another avenue for hydrophobic COF membranes could be their use as adsorptive membranes, which will provide ease of operation and allow continuous processes.
